# Ground-Air

**Published:** 1880-09

**Authors:** 


### GROUND-AIR.

A correspondent, who says that he has seen references to "ground-air" in certain articles on sanitary subjects, asks us to explain to him and other readers of the Journal "the meaning and bearing of the term."

We commonly think of the lower limit of the atmosphere as being the surface of the earth, but this is an error. Every part of the soil not occupied permanently by the "ground-water" (a term which needs no explanation) contains air between its particles, and this air is continually varying both in quanity and quality. As the groundwater rises, it presses on it and forces it out, making the earth expire, so to speak ; when the ground-water falls, a vacuum is formed, which causes an inspiration to take place. The rise and fall changes the air which fills all the interspaces between the solid particles of clay, sand, or rock, which are so situated that they can be affected by the movement. The atmospheric air in the soil is of course altered considerably by the nature of the media to which it is exposed ; but it consists principally of carbonic acid, carburetted hydrogen in small quantities, depending on the soil, marsh gas, sulphuretted hydrogen, and, frequently, other gases from putrefying organic matter. As the ground-water rises it forces out the air, and with it all gases and emanations which are mischievous and unwholesome. This process is greatly assisted by the aspirating effect of a warm house, with no impervious flood to prevent the foul exhalations from being drawn into the dwelling. These are sucked at an almost inconceivable distance from the sides and neighborhood of a dwelling as well as from beneath the actual structure itself. The following case, which was reported by Professor de Chaumont, in the London *Lancet*, affords a good example of the suction power of a house built upon a porous soil :

In December, 1870, Mrs. B., aged 82, her grandson 38, and granddaughter 30, lived in a small, four-room semi-detached house at Itchen Ferry, opposite Southampton. The gas company had recently laid down pipes along this road, and immediately opposite the house a siphon was placed. The pipes were sunk deep in the ground, and had been there for some months before the gas was turned on; when laid down first they were tested, and were not again examined. In the mean time a severe frost had set in, and this, no doubt, prevented the escape of gas into the air, and sealed up the surface of the ground. The gas was first turned on on December 22. No particular smell was noticed in the house, and the family went to bed about 10 P.M. On the morning of the 23d, as no one could gain admission to the house, the neighbors became alarmed, and determined on breaking into the place. On the door being forced open, the apparently lifeless body of the younger woman was found in the passage, and she was the only one who eventually recovered. The dead body of the elder woman was found in the front bed room, and the man in the back room. The young woman first noticed the smell, and endeavored to reach the door, but failed and fell down in the passage. On examination, the siphon in the roadway was found cracked, and the soil, which consists of loose gravel, between it and the house was largely charged with gas, which passed under the wall of the house and up through the sitting-room floor, for the smell was strongly marked under the floor-planking and in the cupboard. Both rooms were destitute of the means of ventilation, for the windows were closely shut and the fire-places boarded up and pasted carefully over at all parts with brown paper. The coroner's jury brought in a verdict that the deceased met their deaths from the effects of poisoning by coal gas.

In this case the gas made its way through the ground for a distance of about twenty-five feet, and under the foundation wall of the house. The ground being frozen prevented the escape upwards, and the same condition of things is found in all large cities, where the streets are almost hermetically sealed up by the pavements, which prevent the free exit into the external air of the deleterious gases in the soil. The street gas pipes almost always leak, as any person may satisfy himself by the sense of smell when the streets are dug for repairing or tapping the pipes.

All these extracting forces are of course intensified by the warmth of the house, and especially of the kitchen fire, which is often in the basement, beneath the level of the road or street. External temperature also causes no small movement in this ground-air, promoting its intermixture with the atmospheric acid. The ground (except when covered with snow) gets heated by day and cooled by night, and heated air, we know, always expands.

The ground thus taken up cool air, which it contaminates and exhales when the temperature is raised. In addition to this, we have constant diffusion or interchange taking place between the air above and the air below the surface; and if the former is confined or stagnant both will become foul. In towns especially is the ground-air vitiated by gas and sewer emanations through leaky pipes. Few persons can have passed by an excavation of any part of a street in our cities without having been attracted by other stenches than that of coal gas emanating from the upturned soil, and these, when sealed over by an almost impervious layer, have no free escape except into the basements of our houses. While we are improving our streets by sealing them up with stone, asphalte, or other pavings, it is a matter of serious consideration whether at the same time we are not rendering our houses more unhealthy. The movement of the air in the ground, whether caused by change of temperature or by difference in the level of the ground-water, must take place through the houses. We learn from the experiments of Pettenkofer and others that not only is the amount of carbonic acid in the ground-air exceedingly large, but that the proportion of carbonic acid in ground-air is nearly fifty per cent. more than in ground-water.

From what has been said, it will be evident why the immediate vicinity of graveyards and cemeteries is often unhealthy. Not only is the ground-water contaminated, but the poisonous vapors pass into the air, and even if they do not develop specific disease they certainly add to the average death rate. According to Professor Parke, than whom there can be no better authority on such a point, epidemic diseases introduced under these circumstances assume a specially virulent character, and are exceedingly fatal.

Our friend and other readers to whom this subject may be comparatively new, will see from this brief and familiar presentation of the leading facts concerning it that, in the location of a dwelling, the nature of the ground-air and its possible sources of contamination should be carefully considered. The breathing of our mother earth may vitiate the atmosphere, as the breathing of living creatures does, and we must beware of habitually taking the polluted air into our own lungs.—*Boston Journal of Chemistry.*

